# Implementation of the Australian national hand hygiene initiative

**DOI:** 10.1186/2047-2994-4-S1-O46

**Published:** 2015-06-16

**Authors:** AJ Stewardson, PL Russo, M Cruickshank, ML Grayson

**Affiliations:** 1 Hand Hygiene Australia, Heidelberg, Australia; 2 Australian Commission on Safety and Quality in Health Care, Darlinghurst, Australia

## Introduction

In 2008, the Australian Commission on Quality and Safety in Health Care (ACSQHC) engaged Hand Hygiene Australia (HHA) to implement the National Hand Hygiene Initiative (NHHI), a national approach to hand hygiene (HH) culture change adapted from the WHO Multimodal HH Improvement Strategy.

## Objectives

To achieve sustained improvements in HH performance amongst healthcare workers, reduce healthcare-associated infections, develop an effective education and credentialing system about HH and infection control, and make HH and infection control core business for all healthcare institutions.

## Methods

HHA, the ACSQHC, and jurisdictional authorities collaborated to support healthcare institutions implement the NHHI. At institution level, core components of the NHHI are alcohol-based handrub at the point-of-care, healthcare worker education, and HH auditing with performance feedback using the WHO ‘5 Moments’ methodology. HHA developed an implementation manual, profession-specific e-learning modules, HH auditor training workshop materials, a web-based database for monitoring and reporting HH performance and other resources. Since 2011, the interim national benchmark for HH compliance has been 70%, and aggregate institution-level compliance has been reported publically online. In 2013, implementation of the NHHI became a hospital accreditation requirement.

## Results

The HHA e-learning modules have been completed more than 800,000 times. The number of healthcare institutions submitting HH data increased from 290 in 2009 (262 public and 28 private institutions) to 828 in 2014 (535 public and 293 private). Over the same period, HH compliance increased from 61.8% (95% confidence interval, 61.5–62.0) to 81.9% (95% CI, 81.8–82.0). In 2014, 98% (814/828) of participating institutions met the 70% national benchmark. The incidence density of healthcare-acquired *Staphylococcus aureus* bloodstream infection has decreased in parallel over the same period.

## Conclusion

The NHHI has been a successful national quality improvement program. Key contributors to this success include leadership from the ACSQHC, a standardised national approach with collaboration between federal and jurisdictional authorities, adoption of WHO methodology, incorporation into hospital accreditation, and significant efforts from frontline infection control practitioners.

## Disclosure of interest

None declared.

